# Effects of species diversity on trait expression of the clonal herb *Taraxacum officinale* and its relation to genotype diversity and phenotypic plasticity

**DOI:** 10.1002/ece3.11430

**Published:** 2024-05-16

**Authors:** Francesca De Giorgi, Christiane Roscher, Walter Durka

**Affiliations:** ^1^ Department of Physiological Diversity Helmholtz Centre for Environmental Research – UFZ Leipzig Germany; ^2^ German Centre for Integrative Biodiversity Research (iDiv) Halle‐Jena‐Leipzig Leipzig Germany; ^3^ Department of Community Ecology Helmholtz Centre for Environmental Research – UFZ Halle Germany

**Keywords:** apomixis, biodiversity, clonality, functional traits, genetic diversity, intraspecific trait variation, phenotypic plasticity

## Abstract

Plant species respond to varying plant species diversity and associated changes in their abiotic and biotic environment with changes in their phenotype. However, it is not clear to what degree this phenotypic differentiation is due to genotype diversity within populations or phenotypic plasticity of plant individuals. We studied individuals of 16 populations of the clonal herb *Taraxacum officinale* grown in plant communities of different species richness in a 17‐year‐old grassland biodiversity experiment (Jena Experiment). We collected 12 individuals in each population to measure phenotypic traits and identify distinct genotypes using microsatellite DNA markers. Plant species richness did not influence population‐level genotype and trait diversity. However, it affected the expression of several phenotypic traits, e.g. leaf and inflorescence number, maximum leaf length and seed mass, which increased with increasing plant species richness. Moreover, population‐level trait diversity correlated positively with genotype richness for leaf dry matter content (LDMC) and negatively with inflorescence number. For several traits (i.e. seed mass, germination rate, LDMC, specific leaf area (SLA)), a larger portion of variance was explained by genotype identity, while variance in other traits (i.e. number of inflorescences, leaf nitrogen concentration, leaf number, leaf length) resided within genotypes and thus was mostly due to phenotypic plasticity. Overall, our findings show that plant species richness positively affected the population means of some traits related to whole‐plant performance, whose variation was achieved through both phenotypic plasticity and genotype composition of a population.

## 1 INTRODUCTION

Concerns about the consequences of human‐mediated biodiversity loss have increased the efforts to understand its consequences for ecosystem functioning (Hooper et al., 2005). Genetic diversity, a form of intraspecific variability, is a central component of biodiversity, as it provides the raw material for evolution by natural selection (Dobzhansky, 1938; Hughes et al., 2008). Beyond its well‐known importance for adaptation, it is now appreciated that within‐species genetic variation can also have important ecological consequences for ecosystem processes like primary productivity, resilience against diseases and disturbances and for trophic interactions (Hughes et al., 2008; McGill et al., 2006; Tang et al., 2022).

With the main processes affecting genetic diversity being mutation, drift, migration and selection, it has been hypothesized that also species diversity can influence the level of genetic diversity of a population (Adams & Vellend, 2011; Vellend & Geber, 2005). The diversity and relative abundance of the species composing a community can create environments with different selection regimes to which coexisting populations are exposed, with the consequent possible alteration of their genetic diversity. Specifically, two main hypotheses suggest causal, but opposite effects of species diversity on genetic diversity: species diversity can be a source of diversifying selection, i.e. creating a positive relationship between species diversity and genetic diversity (Adams & Vellend, 2011; Marquard et al., 2009; Vellend & Geber, 2005), or it can lead to stabilizing selection, i.e. resulting in a negative relationship between species diversity and genetic diversity (Van Valen, 1965; Vellend & Geber, 2005). The two hypotheses have different implications for population‐level trait diversity, i.e. variation in ecological and functional traits. Following the first hypothesis, numerous coexisting genotypes would produce diverse phenotypes with different abilities to compete for resources (Hughes et al., 2008; Mulder et al., 2016; Vellend & Geber, 2005). With the second hypothesis, the presence of fewer genotypes could prevent a population from producing diverse phenotypes (Hughes et al., 2008; Mulder et al., 2016), but a high phenotypic diversity could still be achieved through phenotypic plasticity (Noel et al., 2007). Phenotypic plasticity is defined as the ability of a genotype to express different phenotypes in different environments (Pigliucci, 2001), and it can affect many ecologically important traits (Pieruschka & Schurr, 2019; Sultan, 2000). Several studies showed how phenotypic plasticity can be seen as a property of a genotype, which, due to an environmental stimulus, makes the appearance of different phenotypes possible (Pigliucci et al., 2006). Therefore, it is considered as an advantageous feature in changing environments.

In this study, we focus on the expression of phenotypic traits representing the overall plant performance in environments characterized by different plant species diversity. Phenotypic traits of plant species are variable at various organizational levels and due to different underlying mechanisms (Westerband et al., 2021). Different trait values often reflect strategies used by individuals to adjust to their actual abiotic and biotic environment (Suding et al., 2003). For example, previous studies have shown that increasing plant species diversity generally leads to an increase in community biomass production (Roscher et al., 2005); however, individual species differ in their biomass response to species diversity (Lipowsky et al., 2011; Thein et al., 2008). The denser and taller neighbors, typical of high‐diversity communities (Lorentzen et al., 2008), shade smaller plants during their growth. Their response to shade leads to an increase in plant height (to avoid canopy shade) and specific leaf area (SLA) (to tolerate canopy shade) in a species‐rich environment (Bachmann et al., 2018; Lipowsky et al., 2011, 2015). Further leaf traits which could promote plant adjustment to light conditions are leaf greenness (a measure of chlorophyll concentration) and leaf dry matter content (LDMC), but previous studies did not find species diversity effects on the expression of these leaf traits (Bachmann et al., 2018). Leaf nitrogen concentration is an important indicator for photosynthetic carbon gain as it positively correlates with rates of light‐saturated photosynthesis (Anten & Hirose, 2003), but mostly plant community composition, i.e. especially the presence of legumes, rather than species diversity affects leaf nitrogen concentrations (Guiz et al., 2018; Lipowsky et al., 2015). Increased community diversity can also alter plant reproductive efforts. For example, as a consequence of increased plant height due to increasing species diversity, individuals may invest more in inflorescence production (Levins, 1968). However, higher competition may lead plants to invest mostly in vegetative growth, thus allocating fewer resources to reproduction by seeds (Levins, 1968). The trade‐off between vegetative and reproductive growth has been demonstrated in previous studies (Schmidtke et al., 2010) and an overall negative effect of increasing species richness on the proportion of flowering individuals was found (Lipowsky et al., 2011; Roscher, 2008). However, focusing on other important traits for reproduction, such as seed mass and germination rate, Rottstock et al. (2017), found no effects of plant community diversity, while (Lipowsky et al., 2012) observed a positive effect of increasing plant species diversity on average germination rates of *Taraxacum officinale* seeds.

The variation in phenotypic traits among individuals in a population (Albert et al., 2010; Jung et al., 2010; Siefert, 2012) influences its ability to respond to the abiotic environment and biotic interactions of a community (Fridley et al., 2007; Fridley & Grime, 2010), as well as its effects on ecosystem processes (Crutsinger et al., 2006; Hughes et al., 2008). However, it is still not clear to what degree the observed phenotypic variation in response to plant species diversity is due to genotype composition of populations or phenotypic plasticity of plant individuals. To disentangle the role of these two sources of trait variation, we investigated population responses, i.e. changes in population‐level trait means, to plant species diversity in 17‐year‐old communities of a grassland biodiversity experiment, using *T. officinale* as a model species. *Taraxacum officinale* is an apomictic species aggregate in the study area, which stores high genotypic variability (Kirschner et al., 2016; Preite et al., 2015). Apomixis in the species allows the study of phenotypic variation on several individuals with the same genetic background. Therefore, this species is suitable to disentangle the role of the two candidate sources of variation. We hypothesized that

1. Species richness has an effect on genotypic richness.
2. Species richness has an effect on population‐level trait means and population‐level trait diversity.
3. Genotypic richness has an effect on population‐level trait diversity.
4. Both genotypic composition and phenotypic plasticity explain the species richness effect on population‐level trait means.

## 2 MATERIALS AND METHODS

### 2.1 Study site – Jena experiment

The Jena Experiment is a long‐term biodiversity experiment located in the floodplain of the Saale River near Jena (Thuringia, Germany, 50°55′ N, 11°35′ E, 130 m a.s.l.) (Roscher et al., 2004). It was established in 2002 on what had been a highly fertilized arable field from the early 1960s until 2000, used to grow vegetables and wheat. The soil on the site is a Eutric Fluvisol (FAO‐Unesco 1997), whose texture varies from sandy loam to silty clay with increasing distance from the river. Therefore, the experiment was organized in four blocks parallel to the riverside according to these soil characteristics (Roscher et al., 2004). A pool of 60 species was selected to be part of this experiment, and later classified into four functional groups: grasses, small herbs, tall herbs and legumes. The species pool was used to create different mixtures crossing in a near‐orthogonal design the experimental factors species richness from 1 to 60 (1, 2, 4, 8, 16, or 60 species) and functional group number (1, 2, 3, or 4 functional groups). Each species‐richness level had 16 replicates with different species compositions, except the 16‐species mixtures with 14 different replicates, and the 60‐species mixture with 4 replicates, resulting in a total number of 82 plots. Plot size was 20 × 20 m, which was reduced to 6 × 6 m in 2010 (Weisser et al., 2017). For more information, see (Roscher et al., 2004). In early May 2002, seeds were sown with a density of 1000 viable seeds per m^2^ with equal proportions for all species in a mixture. Seeds were purchased from a commercial supplier specialized in seeds of regional origin (Rieger‐Hofmann GmbH, Blaufelden‐Raboldshausen, Germany). According to the typical management of extensive hay meadows of the region, plots were mown twice per year (in June and September) and the mown material was removed. The experiment did not receive any fertilization. All plots were weeded two to three times per year to remove all species not sown into a particular plot and keep the sown species combinations. Thus, weeding did not manipulate the originally sown species mixture. The realized species richness remained highly correlated to the sown species richness even after several years (Weisser et al., 2017) although community assembly processes and different response of species to environmental variation resulted in a temporally varying species abundances. Our study included also three locations in the close surroundings of the Jena Experiment. These were two species‐rich extensively used hay meadows north and south of the field site and a ruderal grassland located near the street running west of the field site. The east margin of the field site was not sampled because it is directly adjacent to the Saale River.

### 2.2 Study species: *Taraxacum officinale*

**FIGURE 1:**
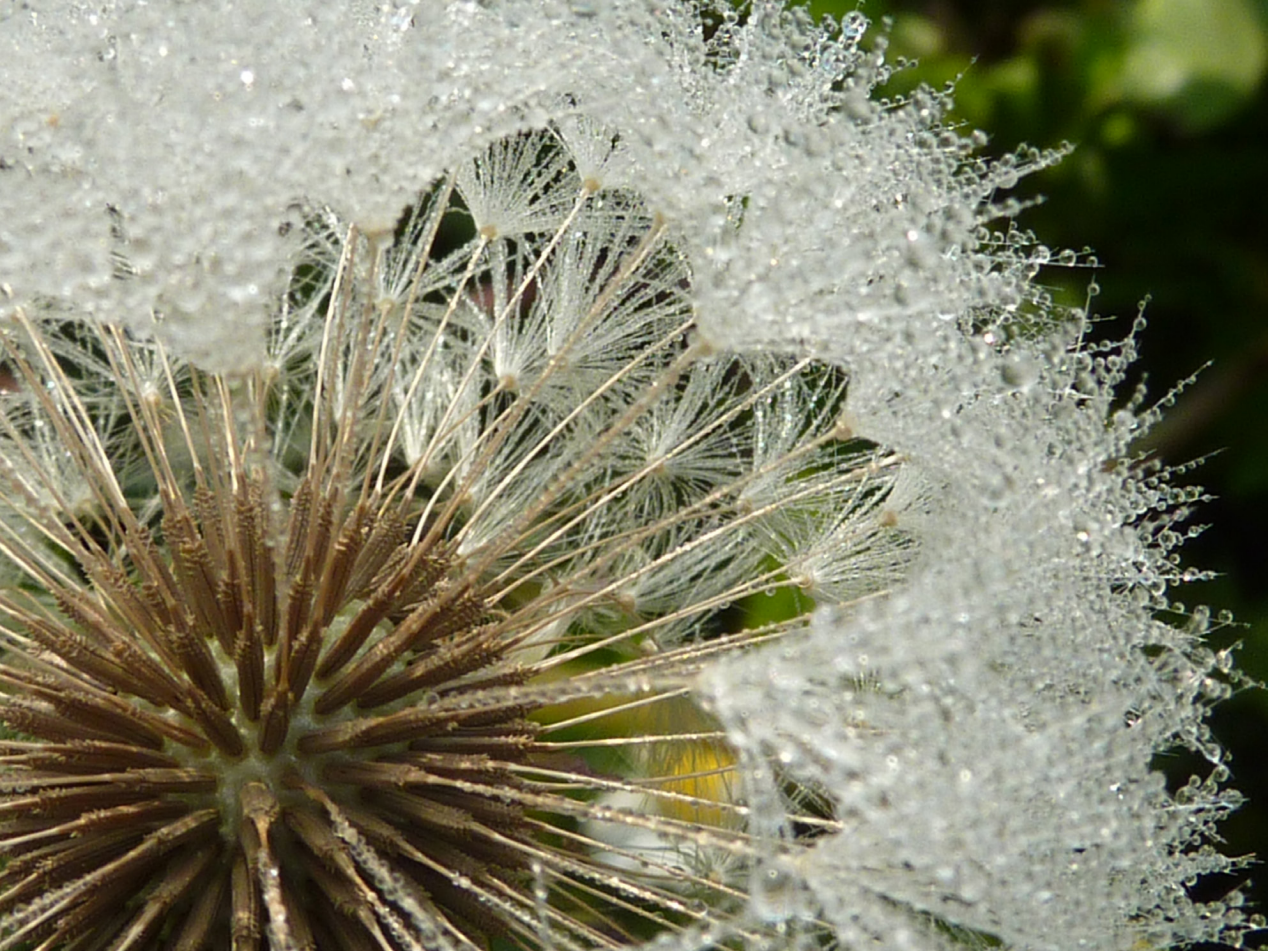
Image of *Taraxacum officinale* infructescence.

*Taraxacum* is a pan‐global genus originated in the temperate regions. *Taraxacum officinale* (L.) Weber ex F.H. Wigg. (syn. *Taraxacum* sect. *Ruderalia*, common dandelion) is a species aggregate with variable ploidy level, mating system and degree of reproductive isolation (Figure 1). In Europe, it mostly shows a distribution characterized by polyploidy in the North and by diploidy in the South (Van Dijk, 2003). In Central Europe, coexistence of sexual diploids (2× = 16) with apomictic polyploids, which are usually triploids (3× = 24), is quite common (Ozias‐Akins & van Dijk, 2007). Asexual reproduction in *T. officinale* is achieved through meiotic diplosporous apomixis that involves parthenogenetic embryo formation from unreduced egg cells (Koltunow & Grossniklaus, 2003), resulting in clonal seeds, dispersed through wind, but mostly near to the parent plant (Nathan et al., 2008). The usual frequency distribution of *Taraxacum* clones in populations is L‐shaped, meaning that a few common and many rare clones are present (Chaboudez & Burdon, 1995; Menken et al., 1995), a sign of the presence of clonal diversity in apomictic *Taraxacum* populations (Van Dijk, 2003).

### 2.3 Sampling and measurements

*Taraxacum officinale* was part of the sown species combinations in 16 plots of varying species richness (from 2 to 60) in the Jena Experiment (Table S1). Between 5 and 7 May 2019, 12 *T. officinale* individuals with ripe seeds, distant at least one meter from each other and to the plot margin, were chosen in each plot where *Taraxacum* belonged to the sown species combinations. First, one to two inflorescences with ripe seeds were collected and stored in paper bags. Then, the number of leaves and remaining inflorescences were counted and the stretched length of the longest leaf was measured. Leaf greenness, which is an estimate of chlorophyll concentrations assessed by measuring the absorption of two different wavelength (650 and 940 nm) with a portable chlorophyll meter (SPAD‐502 Plus, Konica Minolta), was recorded for each individual by three averaged readings on different young, but fully expanded leaves. Two to three fully developed leaves were sampled and stored in a cooled plastic box with moist tissue paper. For these leaves, fresh mass was weighed after dabbing dry the water‐saturated leaves with tissue paper to remove any water droplets, and leaf area was measured with a leaf area meter (LI‐3000C Area Meter, LI‐COR, USA). Then, the samples were dried at 70°C for 48 h. Dry samples were weighed again to calculate specific leaf area (mm^2^ mg^−1^) as the ratio between leaf area and weight of dry leaf material, and leaf dry matter content, as the ratio between leaf dry mass and fresh mass (mg g^−1^). Leaves were then milled to a fine powder with a ball mill (MM200, Retsch, Haan, Germany). Approx. 10 mg of leaf material from around 20 samples was used to determine nitrogen concentration (mg N g^−1^) with an elemental analyzer (Vario EL cube, Elementar Analysesysteme, Langenselbold, Germany). In this way, we were able to create different models and choose the best one to be applied to the rest of the samples. The optimal NIRS models developed to predict N concentration in the samples had a high coefficient of determination (*r* ^2^ = .97) and were used to calibrate the analyses performed on all the samples using an MPA Fourier Transform near‐infrared (FT‐NIR) spectrometer (Bruker, Billerica, Massachusetts, USA). Infructescences collected in the field were cleaned to get the seeds, which were subsequently counted and weighed to calculate individual seed mass as the ratio between mass and number of seeds. Then, the seeds were stored at −20°C. In June 2020, 50 seeds for each individual were sown in petri dishes filled with mineral sand and germinated for 10 days (16 h day at 20°C, and 8 h night at 14°C) to derive the germination rate. In the field, two further young and fully developed leaves were sampled from each individual and stored in paper bags contained in a plastic bag with silica gel to properly desiccate the samples, in order to be used for DNA extraction and subsequent genotyping. Leaves from additional 10 individuals in each of three different locations in proximity of the experimental field were also sampled, resulting in a total of 30 individuals for genotyping.

### 2.4 Microsatellite genotyping and clone identification

We used microsatellite DNA markers developed for *Taraxacum* (Falque et al., 1998; Vašut et al., 2004) to identify distinct multilocus genotypes. We genotyped 222 individuals at eight highly polymorphic microsatellite loci (Falque et al., 1998), i.e. MSTA44B, MSTA58, MSTA61, MSTA67, MSTA72, MSTA143, MSTA31, MSTA78. DNA was extracted from 10 mg of dry leaf material and extraction followed the manual of DNeasy 96 Plant Kit (Qiagen, Hilden, Germany). Microsatellite DNA amplification was prepared using a multiplex protocol: 1 μL of the forward and reverse primer mix (prepared with 2 μL each of the forward and reverse of each primer and filled up with water until 100 μL were reached), 5 μL of Qiagen Multiplex PCR Master mix (Qiagen, Hilden, Germany) and 2 μL of H_2_O were used for a total of 8 μL of PCR Mix. Polymerase chain reaction (PCR) was performed in a total volume of 10 μL containing 2 μL of DNA and 8 μL of PCR Mix. We ran two different PCR programs. For the primer MSTA72, we ran the following program: denaturation at 95°C for 15 min, followed by 35 cycles at 94°C (30 s), 49°C (60 s) as Tm and 72°C (60 s), and a final extension at 72°C for 10 min. For the rest of the primers, we kept the same program features with a Tm of 55°C. Gene Scan 500LIZ size standard was added to the PCR products, which were then run on an ABI PRISM 3130 Genetic Analyzer (Applied Biosystems, Foster City, California, USA). Genotypes were detected using GeneMapper, version 5.0 (Applied Biosystems). Amplified fragments were manually binned with a threshold determined by peak height distribution.

We analyzed the 222 *T. officinale* samples with the R package *poppr* version 2.9.3 (Kamvar et al., 2014, 2015). We found that the maximum number of alleles was four, potentially due to gene duplication. All the samples had three alleles in at least one locus, suggesting that the population is triploid. We used Bruvo's distance (Bruvo et al., 2004) to assess genetic distance between individual genotypes and defined the threshold of genotypic distance between two samples that are considered the same clone based on the minimum of a bimodal frequency distribution (Figure S1) (Kamvar et al., 2014), as is typically done to distinguish clones (Bienau et al., 2016; Gitzendanner et al., 2012). We used the function poppr::mlg.filter using *farthest neighbor* as clustering method to distinguish multilocus genotypes (MLG), i.e. clones, using the threshold of Bruvo distance = 0.22. This resulted in 62 clones, which were used to calculate the number of observed multilocus genotypes (MLG) in a plot. However, because sample sizes were different between inside (12 samples) and outside (10 samples) the biodiversity experiment, we used eMLG, the expected number of genotypes based on rarefaction, as a measure of genotypic richness. We also calculated Nei's unbiased gene diversity (H_e_) as the average across loci (Nei, 1978), and the Shannon Index of MLG diversity (H′) (Shannon, 1948).

### 2.5 Data analyses

All analyses were performed with R Statistical Software (v4.2.2; R Core Team 2022, http://www.R‐project.org). Linear mixed‐effects models, implemented with the *lmer* function in the *lme4* package (Bates et al., 2015), were used to evaluate our hypotheses. For data recorded at the plot level, the null model contained block as a random effect, while for data recorded at the level of plant individuals, the random effects were block and plot nested in block. Fixed effects were added stepwise to the respective null model. Models were fitted with the maximum‐likelihood method. Likelihood ratio tests were used to assess the statistical significance of the fixed effects.

To test hypothesis 1 whether species richness had an effect on genotypic richness, sown plant species richness was used as a fixed effect. To test for a possible effect on genotype richness of the proximity to neighboring plots that also contained *T. officinale* in the sown species combinations, the physical distance to the closest plot sown with *T. officinale* was entered as a fixed effect. To test hypothesis 2 whether sown species richness had an effect on trait values measured at the plant‐individual level, we extended the random‐effect model containing block and plot with the fixed‐effect sown plant species richness. Moreover, we calculated population‐level trait diversity based on the 12 sampled individuals per plot using Rao's quadratic entropy as a measure of trait diversity (Rao, 1982) as implemented in the package *FD* (Laliberté et al., 2022). We calculated Rao's Q for single traits and for the combination of all measured traits for each population. Then, we used sown species richness as a fixed effect and compared it to the null model with block as random effect to test whether sown species richness had an effect on population‐level trait diversity. To test hypothesis 3 whether genotypic richness had an effect on population‐level trait diversity for multiple and single traits, the model with block as a random effect was extended with genotypic richness as a fixed effect. To test hypothesis 4 whether the variance in population‐level trait means along the species richness gradient was due to genotype composition of a plot or phenotypic plasticity of the same genotype when growing in different plots, we used the variance partitioning following Lepš et al. (2011). Because we did not include the block effect in this analysis, we corrected all measured trait values for block effects. Then, we calculated for each plot the specific plot average using the trait values measured in this particular plot, and the genotype‐mean plot average using mean trait values of the different genotypes across all samples. Finally, phenotypic plasticity, i.e. within‐genotype variability, was calculated as the difference between the specific plot average and genotype‐mean plot average. Subsequently, we run three ANOVAs with specific plot average, genotype‐mean plot average and phenotypic plasticity as response variables and sown plant species richness as explanatory variable to calculate the proportions of variance attributable to these different sources of variation in trait expression and to assess which portion was explained by sown species richness.

To study differences in mean trait values among genotypes and their phenotypic plasticity along the species‐richness gradient, we selected those genotypes which occurred in the field more than ten times and were distributed along the species diversity gradient without biases for certain species‐richness levels (tested with Chi square test). These criteria were met by five common genotypes, corresponding to 43% of the sampled individuals in the biodiversity experiment. Using the reduced dataset containing the individuals of the five common genotypes, we extended the null model stepwise by adding sown plant species richness, genotype identity and their interaction as fixed effects.

## 3 RESULTS

### 3.1 Frequency and diversity of genotypes as related to sown species richness (H1)

In a total of 222 samples of *T. officinale* collected on plots of the biodiversity experiment (*n* = 192) and its vicinity (*n* = 30), we found 62 different clones. The frequency distribution of clones was L‐shaped (Figure S2), indicating a few common and many rare clones. From these, 40 genotypes (= 144 individuals) were found only in the experimental plots, 11 genotypes (= 15 individuals) only outside and 11 genotypes (= 48 and 15 individuals, respectively) were found both on the experimental plots and outside the experimental field. Expected genotypic richness at plot level ranged from 5 to 9, with a mean of ~7 genotypes per plot. The number of genotypes found in the populations outside the experiment ranged from 7 to 8, being thus comparable with the ones inside (Table S1). Sown species richness did not affect genotypic richness (*χ* ^2^ = 0.22, *p* = .636), indicating that a community with many plant species does not enhance nor disfavor a genotype‐rich population of the study species (Figure S3). We did not find any effect of the proximity to another plot containing individuals of *T. officinale* on the number of observed genotypes in a plot (*χ* ^2^ = 0.01, *p* = .927).

### 3.2 Effects of species richness on population‐level trait means and trait diversity (H2)

**FIGURE 2:**
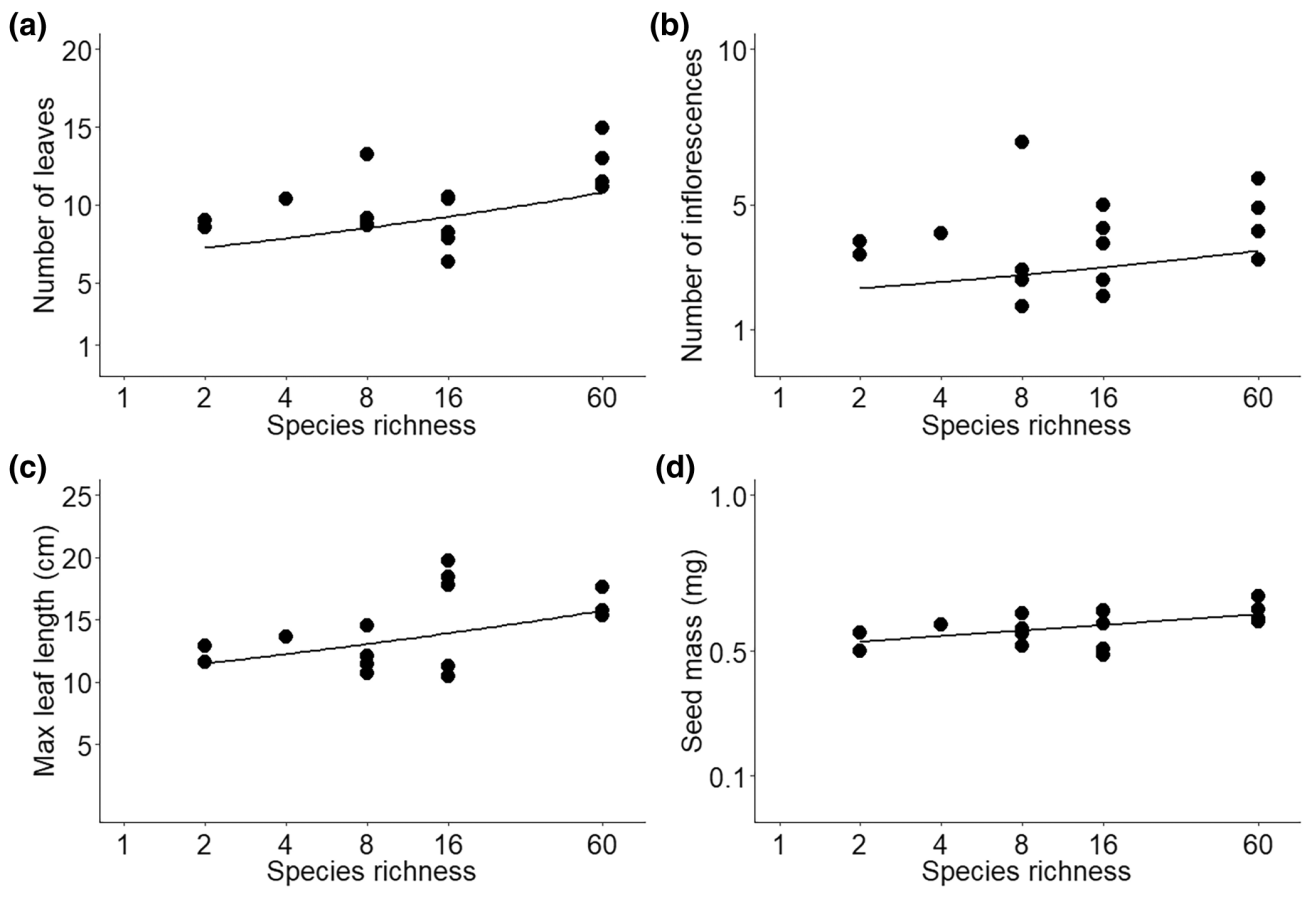
Effects of sown species richness on (a) number of leaves, (b) number of inflorescences, (c) maximum leaf length, and (d) seed mass. Shown are the means across 12 individuals measured for each of the 16 populations of the study (= population means). A black line represents a significant relationship between plant species richness and trait values.

**FIGURE 3:**
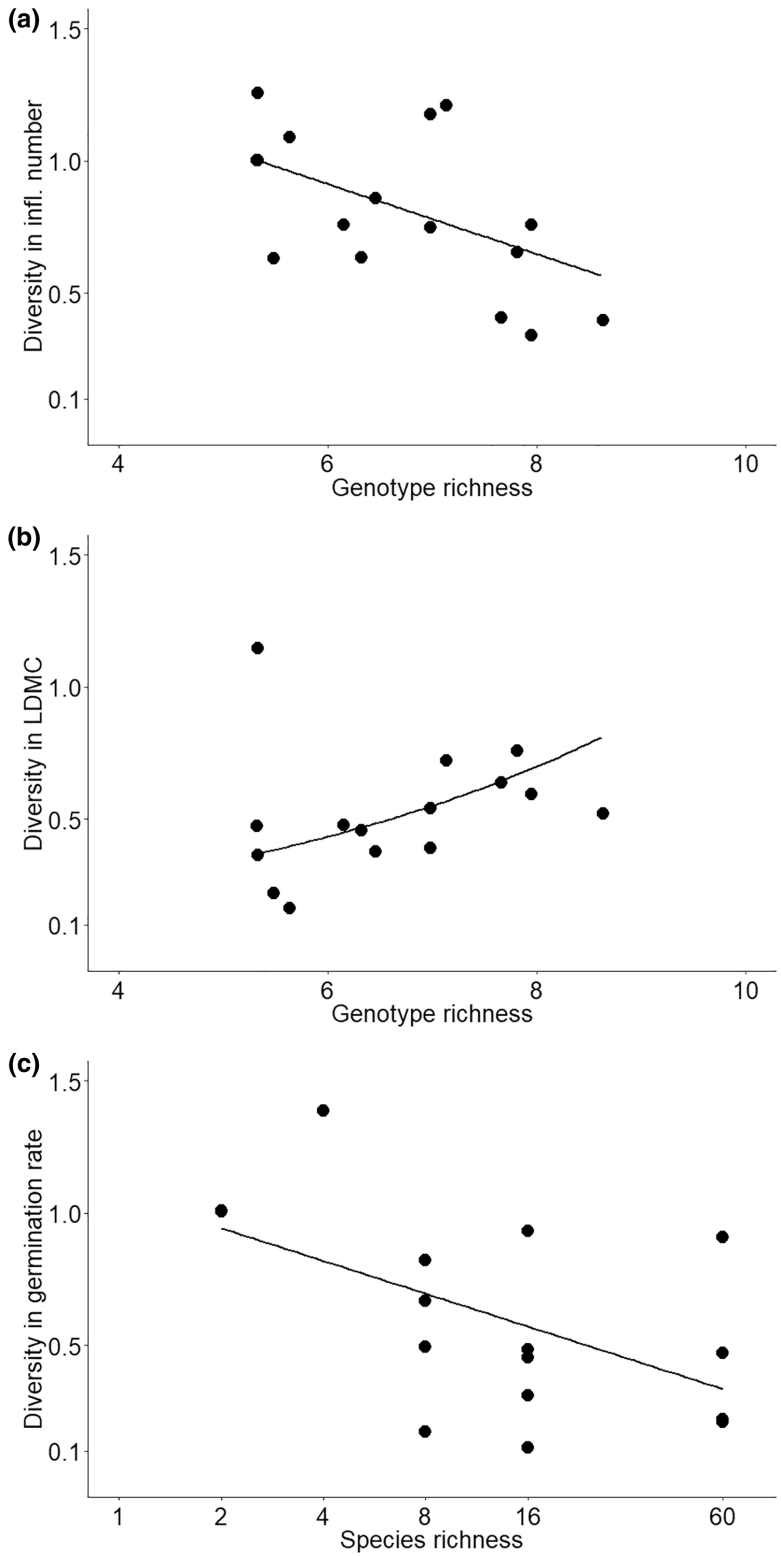
Effects of genotypic richness on (a) population‐level diversity in number of inflorescences, and (b) population‐level diversity in LDMC; effects of species richness on (c) population‐level diversity in germination rate. Population‐level trait diversity was calculated based on the 12 sampled individuals per plot using Rao's quadratic entropy (Rao, 1982). A black line indicates the significant relationship between genotypic/species richness and population‐level trait diversity.

On average, the number of leaves and inflorescences, maximum leaf length and seed mass increased with increasing species richness (Figure 2, Table 1). There was no response to species richness in the other measured traits (SLA, LDMC, leaf greenness, leaf nitrogen concentration, germination rate) (Table 1). Population‐level trait diversity of *T. officinale* for multiple or single traits did not increase with sown plant species richness (Table 1, *χ* ^2^ = 0.61, *p* = .437 for multiple traits), with the exception of the germination rate, which decreased its diversity with increasing plant species richness (Figure 3, Table 1).

**TABLE 1:** Results of linear mixed‐effects models testing effects of sown species richness on population‐level trait means and trait diversity, and the effect of genotype richness on population‐level trait diversity.

Trait	Effects of plant species richness on trait means		Effects of plant species richness on trait diversity		Effects of genotype richness on trait diversity	
	*χ* ^2^	*p*	*χ* ^2^	*p*	*χ* ^2^	*p*
Leaf number	5.51	**.019**	0.20	.656	0.23	.135
Inflorescence number	3.75	**.053**	0.20	.651	6.06	**.014**
Leaf length	4.58	**.032**	0.373	.542	0.06	.801
Seed mass	5.24	**.022**	1.51	.219	0.70	.404
SLA	1.67	.196	1.42	.233	1.99	.158
LDMC	1.67	.197	0.41	.524	3.92	**.048**
Leaf greenness	0.09	.470	0.155	.694	1.42	.233
Leaf nitrogen	0.52	.470	3.53	.060	0.10	.753
Germination rate	1.71	.192	5.54	**.019**	0.26	.611
Multiple traits	–	–	0.61	.437	0.002	.967

*Note*: Shown are *χ* ^2^ and *p*‐values. Significant effects are given in bold.

### 3.3 Effects of genotype richness on population‐level trait diversity (H3)

The increase of population‐level trait diversity combining all measured traits with increasing genotype richness was not significant (*χ* ^2^ = 0.002, *p* = .967). Population‐level diversity of inflorescences number decreased with genotypes richness, while diversity in LDMC increased (Figure 3, Table 1). Population‐level diversity in other measured traits was not affected (Table 1).

### 3.4 Genotype identity and phenotypic plasticity as sources of trait variance and their relation to species richness effects on variance (H4)

**FIGURE 4:**
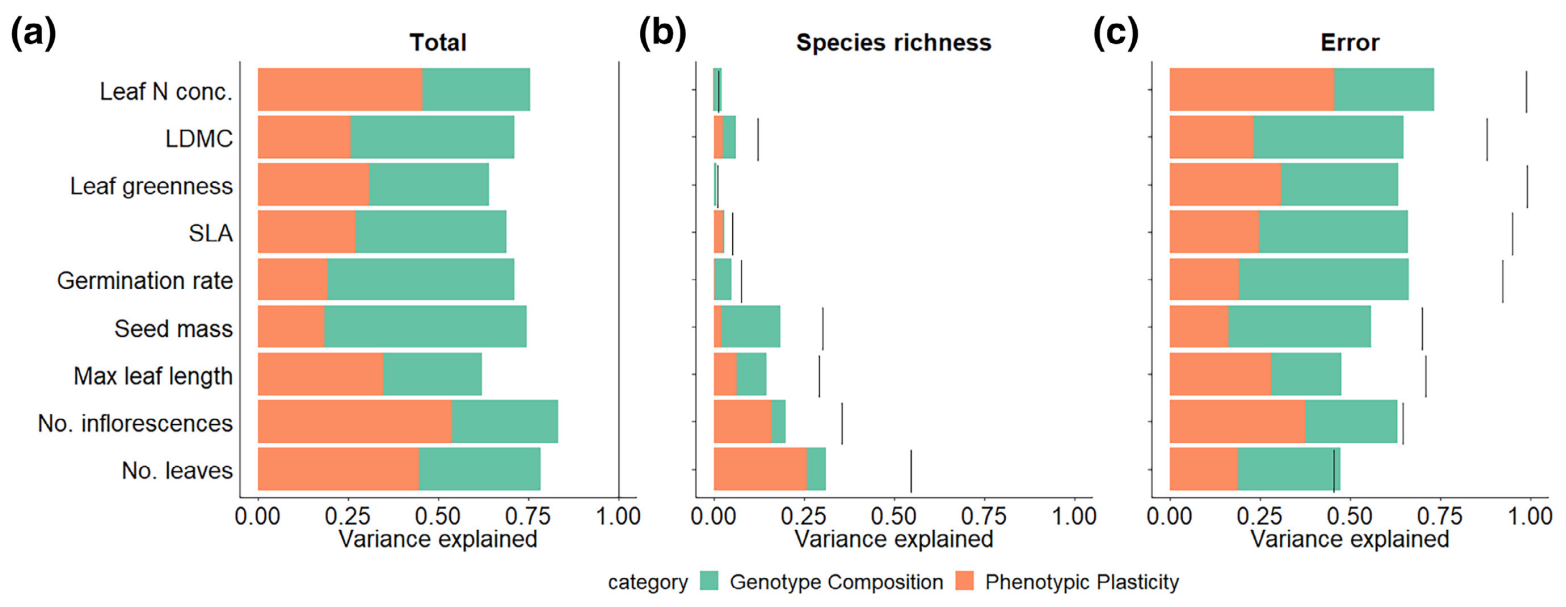
Decomposition of the total variance in the population‐level means of nine studied traits. (a) represents the total trait variation and its component, (b) shows the variation caused by species richness and (c) is the remaining variation that cannot be explained by species richness. Variance caused by genotype composition is shown in green, variance caused by phenotypic plasticity is shown in orange. The black bars represent the total variation for each trait.

Partitioning of variance showed on the one hand that genotype composition was an important component for values of traits such as seed mass, germination rate, LDMC and SLA, where it explained 42%–56% of the total observed variance in population‐level trait means. On the other hand, phenotypic plasticity explained a larger portion of variance (35%–54%) in population‐level trait means for the number of inflorescences, leaf nitrogen concentration, number of leaves and maximum leaf length (Figure 4). In traits that showed a positive response to sown species richness, such as the number of leaves and inflorescences, seed mass and maximum leaf length, between 29% and 55% of variation in population‐level trait means was explained by sown species richness. Only a small portion of variance in population‐level trait means (1%–12%) was explained by species richness in other traits (Figure 4). The contribution of within‐genotype trait variation (i.e. phenotypic plasticity) in explaining the species‐richness effects on population‐level trait means was 26% for the number of leaves and 16% for the number of inflorescences, respectively. On the contrary, genotype composition explained a larger proportion of species‐richness effects on population‐level trait means for maximum leaf length (8%) and seed mass (16%) (Figure 4, Figure S4, Table S2).

### 3.5 Trait differences among common genotypes as affected by genotype identity and phenotypic plasticity (H4)

**FIGURE 5:**
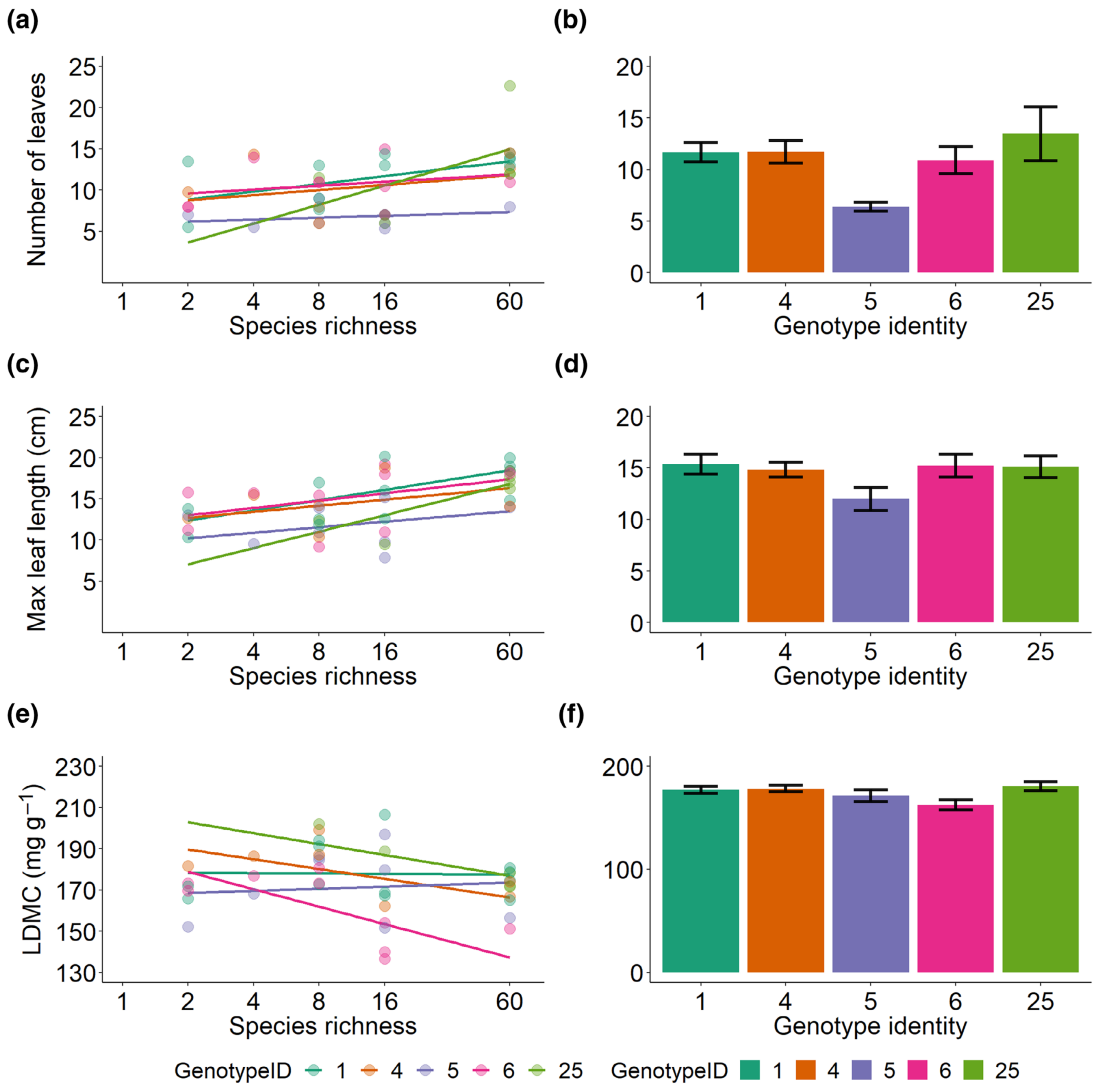
Left panels show the effects of sown species richness on (a) number of leaves, (c) maximum leaf length, and (e) leaf dry matter content (LDMC) for the population‐level means of the five most common genotypes of the study (representing 43% of the full dataset). For number of leaves and maximum leaf length a solid line represents a significant relationship between plant species richness and trait values for each genotype. For leaf dry matter content, a solid line represents a significant interaction between plant species richness and trait values for each genotype. Right panels show the mean trait value (± 1SE) for each genotype across all populations for (b) number of leaves, (d) maximum leaf length and (f) leaf dry matter content (LDMC).

The common genotypes, distributed across all levels of species richness, differed in the expression of most traits, except for the number of inflorescences and seed mass. When analyzing the effects of sown species richness on trait expression of these common genotypes, we found a significant effect for the number of leaves and maximum leaf length (Figure 5). The effect of sown species richness on the expression of these traits did not vary among the genotypes (Table 2). However, the interaction between sown species richness and genotype identity was marginally significant for LDMC, suggesting that distinct genotypes responded differently in their trait expression to increasing plant species richness (Table 2, Figure 5).

**TABLE 2:** Results of linear mixed‐effects models testing for effects of sown species richness, genotype identity and their interactions on trait values of the five most common genotypes (corresponding to 43% of the sampled individuals).

Trait	Species richness		Genotype identity		Species richness × genotype identity	
	*χ* ^2^	*p*	*χ* ^2^	*p*	*χ* ^2^	*p*
Leaf number	6.32	**.012**	12.19	**.016**	3.04	.551
Inflorescence number	0.81	.367	2.29	.682	6.10	.192
Leaf length	6.43	**.011**	14.63	**.006**	3.01	.557
Seed mass	2.82	.093	7.48	.112	6.08	.193
SLA	0.45	.501	21.31	**<.001**	56.24	.182
LDMC	0.659	.417	14.61	**.006**	9.98	**.041**
Leaf greenness	1.14	.287	14.68	**.005**	3.17	.529
Leaf nitrogen	0.026	.872	13.80	**.008**	1.60	.809
Germination rate	3.10	.078	33.60	**<.001**	1.80	.772

*Note*: Shown are *χ* ^2^ and *p* values. Significant effects are given in bold.

## 4 DISCUSSION

### 4.1 Frequency and diversity of genotypes as related to sown species richness

Genotyping and clone identification showed that the 16 experimental *T. officinale* populations of this study were composed of a large number of distinct genotypes. This is in line with various studies from other regions that found high intrinsic genotypic variability in non‐experimental apomictic dandelion populations (Lyman & Ellstrand, 1984; Preite et al., 2015; Solbrig & Simpson, 1974; Vavrek, 1998). An important source of intraspecific variation in apomictic lineages of *T. officinale* is somatic variation (Kirschner et al., 2016; Majeský et al., 2012). This, together with genotyping error, need to be considered when studying clonal apomictic lineages (Gitzendanner et al., 2012). Thus, our results on clonal diversity in *T. officinale* are comparable to previous studies when considering the number and proportions of detected multilocus genotypes (Preite et al., 2015). Indeed, the distribution of all clones found in our study was L‐shaped (Figure S2), consisting of a few common and many rare genotypes, confirming observations of Chaboudez and Burdon (1995) or Menken et al. (1995). Among the 62 identified genotypes, 40 genotypes (67%) were present only in the experimental population, most likely representing genotypes originally sown. Regarding the 11 genotypes (18%) shared between populations within and outside of the biodiversity experiment, we cannot exclude that they originated outside the experimental field. However, although *T. officinale* seeds have the potential for long distance dispersal, most of them are dispersed only near to the mother plant, specifically, 99.5% of seeds are dispersed less than 10 m (Soons & Ozinga, 2005; Tackenberg et al., 2003). This could explain the absence of any effect of proximity of plots containing *T. officinale* individuals on the number of observed genotypes. Therefore, while we cannot totally exclude migration of seeds among plots or from external populations, which could obliterate selective effects of the species diversity gradient created in the Jena Experiment, we consider plot‐level populations to be largely unaffected from neighboring plots. According to competing hypotheses, species diversity is expected to influence genetic diversity either favoring a few dominant genotypes, the ones able to face interspecific competitors (Van Valen, 1965; Vellend & Geber, 2005), or, on the contrary, supporting the establishment of numerous genotypes (Harper, 1977; Vellend & Geber, 2005). In contrast to these hypotheses, we did not find any effect of sown plant species richness on genotypic richness, thus rejecting our initial hypothesis (H1). A possible explanation to this result could be the reproductive mode of *Taraxacum officinale* and its consequences for genetic diversity (Ozias‐Akins & van Dijk, 2007). Indeed, for the apomictic, non‐recombining, lineages in the Jena experiment, phenotypic plasticity could be an important feature to respond to environmental variation (Noel et al., 2007). As seen in previous studies (Noel et al., 2007), populations which cannot rely on variability produced by genetic diversity have a large proportion of variability in traits caused by phenotypic plasticity. Therefore, due to the low possibility of recombination, *T. officinale* could use phenotypic plasticity as a first and fast response to selective environmental pressures, leaving genetic diversity less influenced by them.

### 4.2 Effects of species and genotype richness on population‐level trait diversity

Contrary to our hypothesis (H2), we did not find an effect of increasing species richness on population‐level trait diversity when considering multiple or single traits, with the exception of germination rate, which decreased its diversity. So far, studies carried out on trait diversity in biodiversity experiments mostly focused on interspecific trait variation. This is indeed an important mechanism facilitating the coexistence of different species in diverse communities (Roscher et al., 2018; Silvertown, 2004). Our study aimed to understand how different species richness could structure the population‐level intraspecific trait diversity. As already discussed, we did not find a significant relationship between species richness and genotype richness, which is also reflected in most of the population‐level trait diversity. Although we found plant species richness effects on population‐level mean traits, different individuals within the populations mainly followed the same patterns. Similar results were found in a study by Roscher et al. (2015), which focused on intraspecific variation in light acquisition‐traits of seven legume species in monocultures and a mixture with high plant species diversity. While traits like shoot height and stretched shoot length increased at higher plant species diversity, their diversity mostly depended on species identity (Roscher et al., 2015). We also found that in plots rich of different genotypes, population‐level trait diversity calculated across multiple traits did not increase, while, when considered as single traits, diversity in inflorescences number decreased and diversity in LDMC increased, partly confirming our hypothesis (H3). In our study, the increasing population‐level trait diversity for LDMC with increasing genotypic richness could be due to the fact this trait might be strongly genetically determined. Thus, its expression would mostly rely on standing genetic variation. On the contrary, the decreased trait diversity in inflorescences number with increasing genotypic richness might be a sign of the weak genetic control on this trait. This is supported by other results of this study, showing how inflorescence numbers responded to increasing species richness and how most of its variance was explained by phenotypic plasticity.

### 4.3 Effects of species richness and genotype identity on phenotypic trait expression

In recent years, the ecological importance of intraspecific variation in phenotypic trait expression is increasingly appreciated. Investigating its relation to genetic diversity, several experimental studies showed how genotype‐rich communities can benefit of higher productivity or fitness (Crutsinger et al., 2006; Fridley & Grime, 2010; Hughes et al., 2008; Tang et al., 2022). In our study, we considered nine phenotypic traits, and we measured them in 16 populations of *T. officinale* along the species diversity gradient of the Jena Experiment. We also analyzed these traits separately for a smaller part of the dataset, comprising five genotypes which were abundant across all populations. The common genotypes differed significantly in the values of these traits, with the exception of number of inflorescences and seed mass. This could be a sign that, for these common genotypes, most of the traits are strongly under genetic control and probably the options to vary in their phenotypes due to phenotypic plasticity are low. Anyway, the number of leaves and leaf length both increased with increasing species richness, both at the level of population‐level mean traits and for the common genotypes. Previous studies already observed how traits related to performance respond to increasing species richness either positively or negatively, mostly depending on the characteristics of the single species (Lipowsky et al., 2011; Thein et al., 2008). For example, the height reachable by a species can influence the probability of being overshadowed by taller neighboring species (Thein et al., 2008). For our study, measurements were taken in early May, which is early enough in the growing season for the vegetation to be still not very dense and tall even in highly diverse communities. Therefore, we argue that individuals were most likely growing in an environment providing the advantages of a diverse community, like dilution of pathogens, greater complementarity in the acquisition of above and below‐ground resources (Kulmatiski et al., 2012; Lorentzen et al., 2008), together with a still comparatively low level of competition for light. This could have enhanced the performance of the individuals in species‐rich plots, which had more leaves and grew taller. Supporting this evidence, traits associated with light acquisition like SLA, LDMC and leaf greenness, which have previously shown to respond to increasing species richness (Bachmann et al., 2018; Lipowsky et al., 2015), showed no respective response in our study. In line with the findings of Gubsch et al. (2011), we also did not find an effect of increasing species richness on leaf nitrogen concentrations. It has been shown that the presence of legumes can positively influence leaf nitrogen concentration of neighboring plants (Gubsch et al., 2011), but this was not the case in our study (*χ* ^2^ = 0.35, *p* = .550). Considering traits related to reproduction, such as number of inflorescences, seed mass and germination rate, we found a positive effect of species richness for the first two. In particular, when considering all sampled individuals, the population‐level means for number of inflorescences and seed mass increased in response to species richness. This is in line with findings of Lipowsky et al. (2012), who found a positive effect of species richness on investment in reproductive traits in *T. officinale*, in particular for germination rates and number of produced seeds. Obviously, growth and reproduction of *T. officinale* take place during a seasonal niche with low competition, where possible higher niche complementarity or lower pathogen pressure (Kulmatiski et al., 2012; Roscher et al., 2011) results in a higher performance of the individuals, which is not only reflected in their increased size, but also a greater investment in reproductive traits. For the most common genotypes we found that the genetic control on the number of inflorescences might be low, in favor of a greater role of phenotypic plasticity instead. Germination rates, however, seemed to be under high genetic control, with a no response to species richness. Seed mass is an extremely important ecological trait, whose size is associated with dispersal ability and success in seedling establishment (Westoby et al., 2002); high intraspecific variability in seed size along environmental gradients has been observed, but the difficulty in distinguishing between variation caused by genetic components or phenotypic plasticity persists (Lalonde & Roitberg, 1989; Völler et al., 2012; Wolfe, 1995). In our study, the causes of variation in seed mass are difficult to interpret. First, the large number of identified genotypes does not allow enough replicates to occur along the species diversity gradient and to directly test the amount of phenotypic plasticity for each genotype. Moreover, through our analyses, we found some consistent patterns in the sources of variation of the expression of some traits. This allowed us to separate the causes of the observed variation for traits like inflorescences numbers, germination rate and LDMC. However, the non‐consistent results we obtained when analyzing seed mass did not allow us to disentangle its main source of variation. One possibility explaining our results could be that the selective pressures typical of high diversity communities favor the establishment of genotypes with certain characteristics (larger size, greater investment in reproduction), but we cannot rule out that these genotypes have a high phenotypic plasticity that shapes their phenotype in the high‐diversity communities (H2, H4).

### 4.4 Genotype identity and phenotypic plasticity as sources of trait variation and their relation to species richness effects

As specified above, our results did not show an effect of species richness on genotype richness. Nevertheless, we could test whether the observed trait variation, caused by species richness, was achieved through phenotypic plasticity or genotype composition. In order to persist, plant populations require the ability to cope with environmental variation. Several studies showed how sexual species resort to phenotypic plasticity (at the individual level) and genetically based adaptation (at population level) to maintain fitness (Wilschut et al., 2016). Our analysis partitioning the observed variation in phenotypic trait expression into variation attributable to genotype composition of the populations and phenotypic plasticity showed a high amount of variance explained by genotype composition in most traits, in particular for seed mass, germination rate, LDMC and SLA. Given the fact that we found a large number of rare genotypes which were thus not found at different levels of plant species diversity, we cannot rule out that their unique trait expression is also attributable to phenotypic plasticity in response to increasing plant species richness. Phenotypic plasticity explained anyway a large amount of the total variance for the number of leaves and inflorescences, leaf nitrogen concentration and maximum leaf length. Trait variation along the species‐richness gradient again was either better explained by phenotypic plasticity (number of leaves and inflorescences) or by genotype composition (leaf length, seed mass). Therefore, both genotype composition and phenotypic plasticity are key sources of intraspecific variation and their role and relative importance is different for the different traits analyzed as we hypothesized (H4).

## 5 CONCLUSIONS

Contrary to our hypotheses, population‐level genotype and trait diversity, studied in 16 populations of the common dandelion *T. officinale* in a large grassland biodiversity experiment, were not influenced by species richness. Some of the studied traits, those associated with plant performance, showed a positive response to increasing species richness. Obviously, our study species uses a temporal niche in the growing season for its main growth and investment in reproduction by seeds, which is especially favored in species‐rich plant communities and allowed the plants to reach a higher performance there. Concerning the role of phenotypic plasticity and genotype composition in explaining the observed variation in phenotypic traits, their importance was different for different traits analyzed. To better disentangle their role, an experiment reproducing multiple times the same genotypes in common environments would be needed to clearly separate the phenotypic plasticity and genotype effects on trait variation.

## AUTHOR CONTRIBUTIONS

**Francesca De Giorgi:** Conceptualization (equal); data curation (lead); formal analysis (equal); investigation (lead); visualization (lead); writing – original draft (lead). **Christiane Roscher:** Conceptualization (equal); data curation (supporting); formal analysis (equal); funding acquisition (lead); investigation (supporting); project administration (lead); supervision (lead); writing – review and editing (equal). **Walter Durka:** Conceptualization (equal); data curation (supporting); formal analysis (equal); funding acquisition (lead); investigation (supporting); project administration (lead); supervision (lead); writing – review and editing (equal).

## CONFLICT OF INTEREST STATEMENT

The authors declare no conflict of interest.

## Supporting information


Appendix S1.

## Data Availability

This work is based on data elaborated by the subproject 10 “Plant trait variation and evolution in the biodiversity‐ecosystem functioning context” of the Jena Experiment. The datatsets are publicy available in the Jena Experiment database (https://jexis.idiv.de/), (https://doi.org/10.25829/BN3X‐TX39, https://doi.org/10.25829/T11X‐MH23).
