# Supplementary material for: Effects of species diversity on trait expression of the clonal herb Taraxacum officinale and its relation to genotype diversity and phenotypic plasticity
Source: Ecol Evol. 2024 May 16;14(5):e11430. doi: 10.1002/ece3.11430 (PMC11099733; doi:10.1002/ece3.11430)

# Supplementary Material

##

Table S1: Summary of the 16 sampled populations of the biodiversity experiment and the external populations including the identity of the sampled plots and their assignment to the experimental blocks, the sown species-richness levels (SR), the number of observed genotypes (MLG), the number of sampled individuals, the number of expected genotypes at the smallest sample size (n = 10) (eMLG) and the identity and frequency of genotypes found in each population.

| Plot | Block | SR | Identity of genotypes | No. of sampled individuals | MLG | eMLG |
| --- | --- | --- | --- | --- | --- | --- |
| B1A01 | B1 | 16 | 1, 5, 6, 7(x2), 11, 26, 27(x2), 34, 36(x2) | 12 | 9 | 7.95 |
| B1A22 | B1 | 60 | 1(x2), 2, 4(x3), 12, 13, 25(x3), 31 | 12 | 7 | 6.32 |
| B2A03 | B2 | 60 | 1, 10, 12(x2), 14(x2), 25(x3), 27(x2), 48 | 12 | 7 | 6.45 |
| B2A06 | B2 | 4 | 4(x3), 5(x2), 6(x2), 11(x3), 29, 50 | 12 | 6 | 5.64 |
| B2A17 | B2 | 8 | 1, 3, 4, 5(x2), 29, 30(x3), 37, 43, 49 | 12 | 9 | 7.82 |
| B2A19 | B2 | 2 | 1(x2), 4(x4), 5, 6, 10, 13, 28, 35 | 12 | 8 | 6.98 |
| B3A14 | B3 | 60 | 1(x5), 5, 18, 25(x3), 35, 46 | 12 | 6 | 5.33 |
| B3A16 | B3 | 16 | 6, 8(x2), 9(x6), 17, 38, 39 | 12 | 6 | 5.32 |
| B3A19 | B3 | 2 | 1(x2), 6, 18, 20, 26(x5), 29, 42 | 12 | 7 | 6.15 |
| B3A24 | B3 | 16 | 4, 6(x2), 13, 16, 28(x3), 29, 32(x2), 40 | 12 | 8 | 7.14 |
| B4A01 | B4 | 60 | 1, 4(x4), 6, 10, 24, 31, 33(x2), 34 | 12 | 8 | 6.98 |
| B4A02 | B4 | 16 | 1, 5(x2), 18, 19(x2), 21, 25(x2), 31, 41, 60 | 12 | 9 | 7.95 |
| B4A06 | B4 | 8 | 1(x3), 8(x4), 10(x2), 28, 30, 32 | 12 | 6 | 5.48 |
| B4A08 | B4 | 8 | 5, 6, 12, 18, 19(x2), 20, 21, 25(x2), 26, 37 | 12 | 10 | 8.64 |
| B4A16 | B4 | 8 | 1(x4), 2, 4, 6, 18, 22, 23, 44, 47 | 12 | 9 | 7.67 |
| B4A18 | B4 | 16 | 1(x5), 5(x3), 14, 15, 27, 45 | 12 | 6 | 5.33 |
| Meadow (north) | / | / | 1(x3), 2, 18, 19, 22, 23, 53(x2) | 10 | 7 | 7.00 |
| Ruderal grassland (west) | / | / | 3, 20(x2), 21(x2), 54, 55, 57, 59, 61 | 10 | 8 | 8.00 |
| Meadow (south) | / | / | 24, 51, 52(x3), 54, 56, 58, 60, 62 | 10 | 8 | 8.00 |

Table S2: Summary table showing the statistics of the relationship between plant species richness and genotype composition/phenotypic plasticity and their effects on trait variance. Two ANOVAs were run using genotype-mean plot average and phenotypic plasticity as response variables and sown plant species richness as explanatory variable. Shown are F and P values of both the analyses. Significant effects are given in bold.

|  | Genotype Composition | | Phenotypic Plasticity | |
| --- | --- | --- | --- | --- |
| Trait | F | P | F | P |
| Leaf number | 2.66 | 0.126 | 18.88 | **< 0.001** |
| Inflorescence number | 2.06 | 0.173 | 6.02 | **0.028** |
| Leaf length | 5.88 | **0.030** | 3.24 | 0.093 |
| Seed mass | 5.93 | **0.029** | 1.56 | 0.232 |
| SLA  LDMC  Leaf greenness  Leaf nitrogen concentration  Germination rate | 0,16  1.28  0.35  1.03  1.35 | 0.690  0.280  0.560  0.330  0.260 | 1.37  1.44  0.01  0.02  0.30 | 0.260  0.250  0.910  0.890  0.600 |

Figure S1: Results of different clustering methods (Nearest neighbor, UPGMA, farthest neighbor) used to distinguish multilocus genotype (MLG) clones, and the histogram based on farthest neighbor clustering. The threshold of Bruvo distance = 0.22 resulted in 62 clones on a total of 222 individuals.


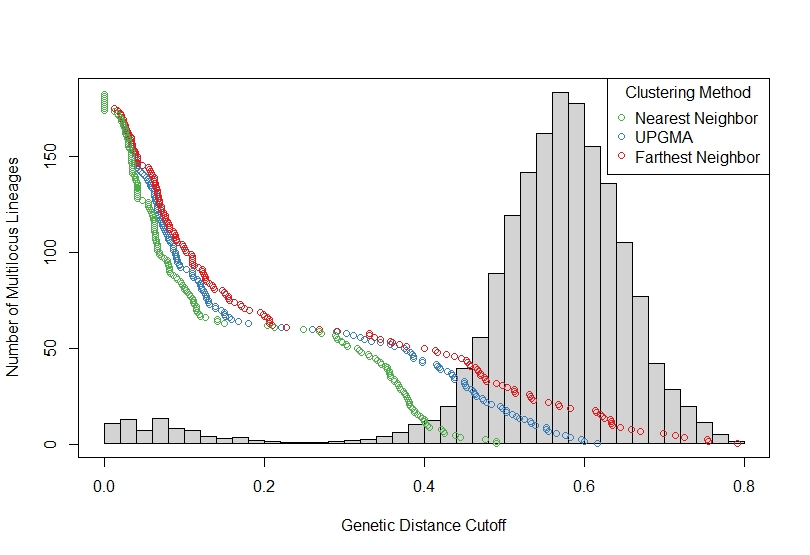


Figure S2: Frequency and distribution of the clones found in our study, inside and outside the experimental field. The distribution was L-shaped, thus consisting of a few common and many rare genotypes.


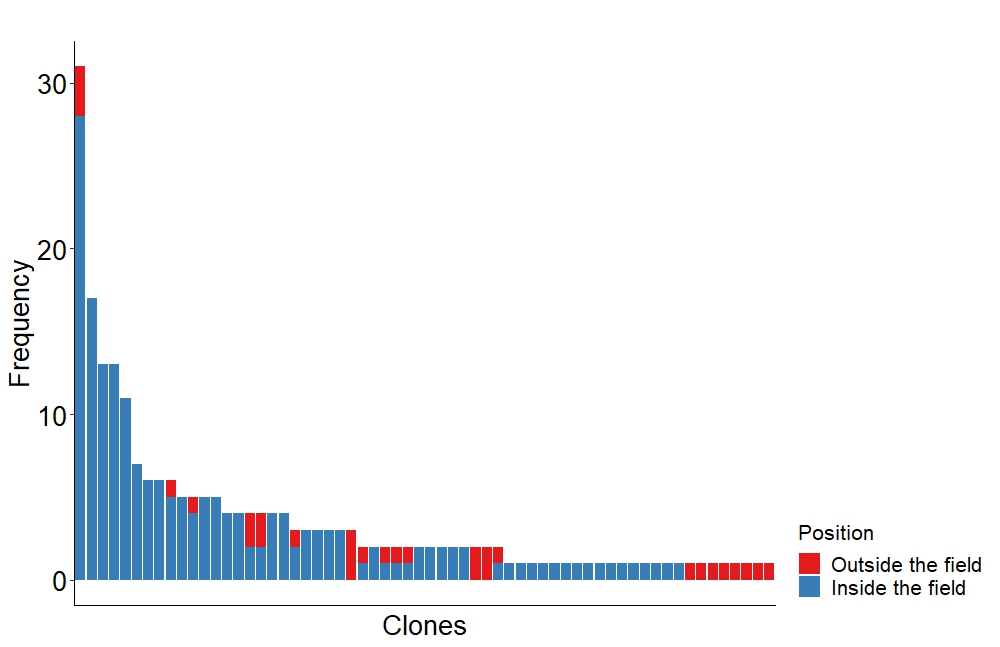


Figure S3: Effects of sown species richness on population-level genotype richness. The absence of a black line indicates that there was no significant species-richness effect.


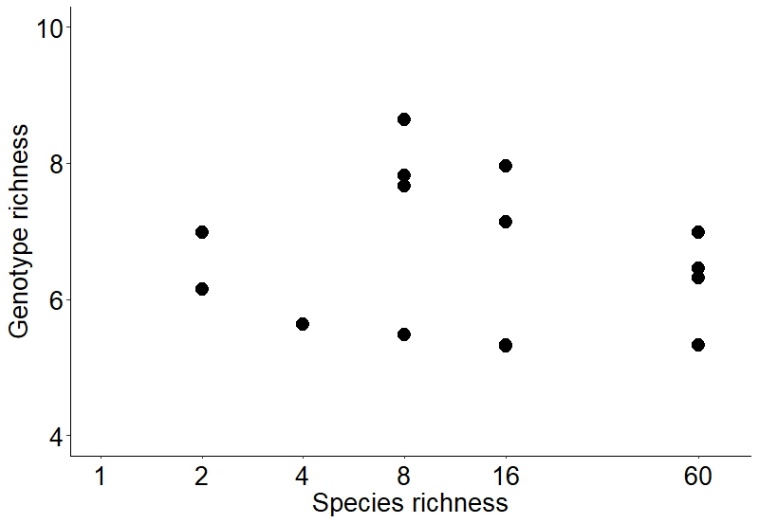


Figure S4: Relationship between plant species richness and genotype composition/phenotypic plasticity and their effects on trait variance. Solid lines represent a significant effect of species richness, dashed lines indicate non-significant effects. The effects of phenotypic plasticity significantly increase with increasing species richness for (A) number of leaves, and (B) inflorescences, while the effects of genotype composition significantly increase with increasing species richness for (C) maximum leaf length, and (D) seed mass.

**
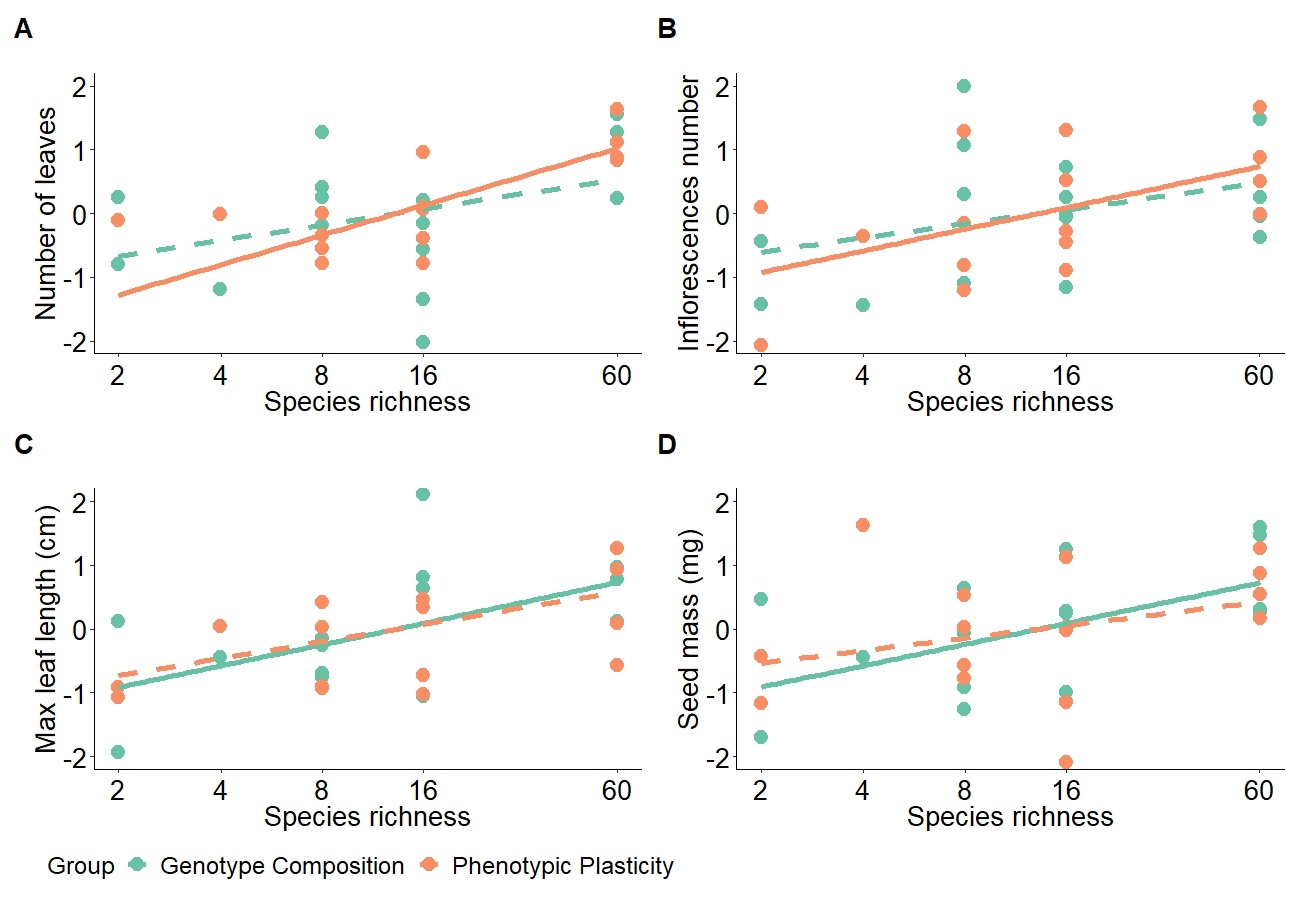
**

Figures S5: Left panels show the effects of sown species richness on (A) germination rate, (C) specific leaf area (SLA), (E) leaf greenness and (G) leaf nitrogen concentration (N_Leaf_) for the population-level means of the five most common genotypes of the study. The relationships are not significant. In the right panels, mean trait value (± 1SE) is represented for each genotype across all populations for (B) germination rate, (D) specific leaf area (SLA), (F) leaf greenness, and (H) leaf nitrogen concentration (N_Leaf_).


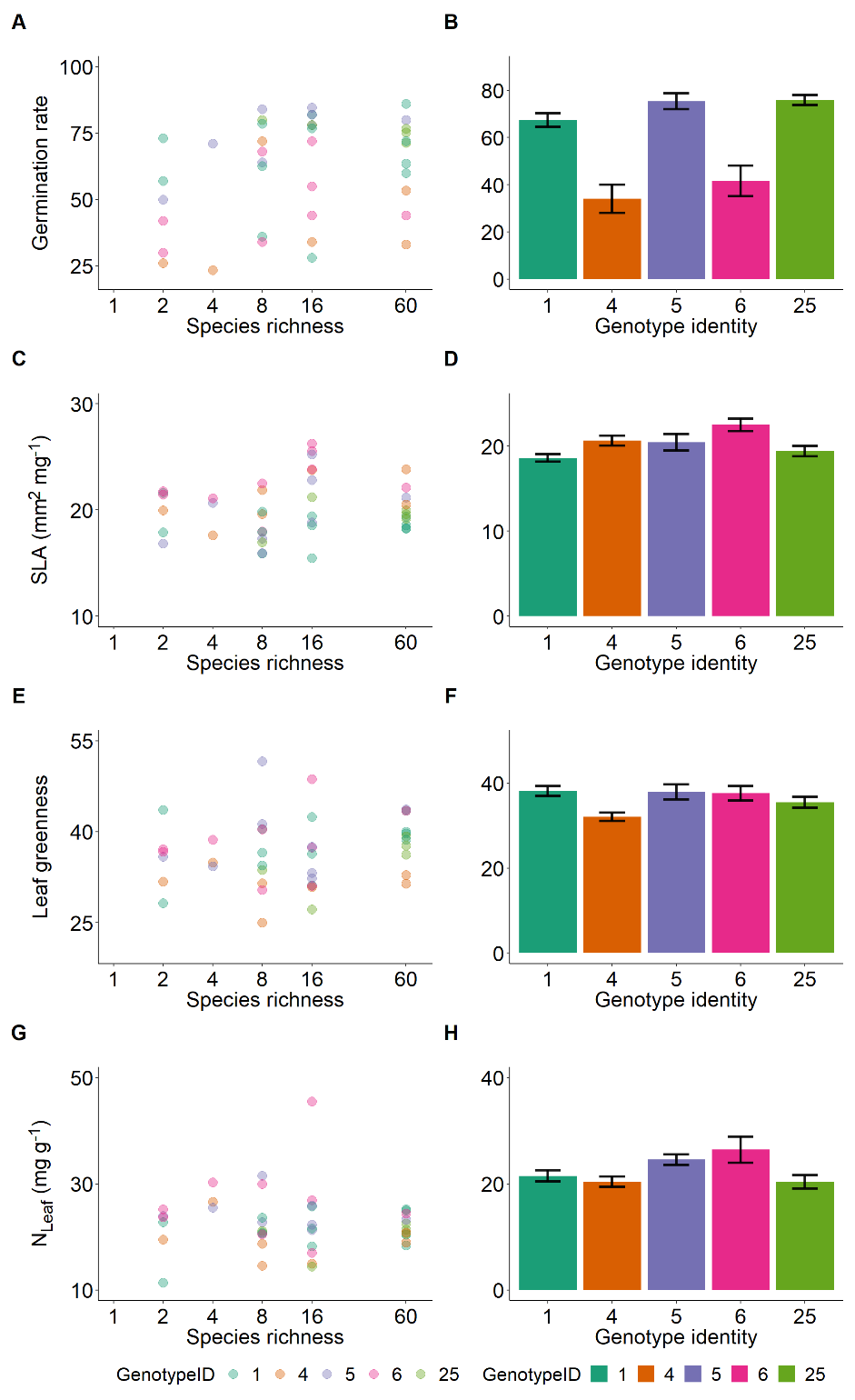

Supplement: Supplementary file 1 — Appendix S1. [file ECE3-14-e11430-s001.docx]
